# Seroprevalence of SARS‐CoV‐2 infection in the general population of Nepal during the first and second generalized waves of the COVID‐19 pandemic—2020–2021

**DOI:** 10.1111/irv.13234

**Published:** 2023-12-27

**Authors:** Krishna Prasad Paudel, Reuben Samuel, Runa Jha, Basu Dev Pandey, Chathura Edirisuriya, Nebin Lal Shrestha, Pradip Gyawali, Amrit Pokhrel, Lilee Shrestha, Ram Kumar Mahato, Shaikh Shah Hossain, Govindakarnavar Arunkumar, Anindya Sekhar Bose, Meghnath Dhimal, Dipendra Gautam, Subash Neupane, Nishant Thakur, Saugat Shrestha, Nirajan Bhusal, Priya Jha, Binod Prasad Gupta, Rajan Bikram Rayamajhi, Koshal Chandra Subedi, Shashi Kandel, Mukesh Poudel, Lila Bikram Thapa, Guna Nidhi Sharma, Allison Eugenio Gocotano, Avinash K. Sunny, Rabin Gautam, Deepak Raj Bhatta, Bal Krishna Awale, Bhola Roka, Hemant Chandra Ojha, Phanindra Baral, Mahendra Dhose Adhikari, Guna Raj Lohani, Mahendra Shrestha, Dipendra Raman Singh, Laxman Aryal, Rajesh Sambhajirao Pandav, Roshan Pokhrel

**Affiliations:** ^1^ Epidemiology and Disease Control Division, Department of Health Services Ministry of Health and Population Kathmandu Nepal; ^2^ WHO Country Office for Nepal Kathmandu Nepal; ^3^ National Public Health Laboratory Ministry of Health and Population Kathmandu Nepal; ^4^ DEJIMA Infectious Disease Research Alliance (DIDA) Nagasaki University Nagasaki Japan; ^5^ Central Bureau of Statistics National Planning Commission Kathmandu Nepal; ^6^ National Health Research Council Kathmandu Nepal; ^7^ Ministry of Health and Population Kathmandu Nepal

**Keywords:** COVID‐19, Nepal, pandemic, SARS‐CoV‐2, seroprevalence

## Abstract

Few seroprevalence studies have been conducted on coronavirus disease (COVID‐19) in Nepal. Here, we aimed to estimate seroprevalence and assess risk factors for infection in the general population of Nepal by conducting two rounds of sampling. The first round was in October 2020, at the peak of the first generalized wave of COVID‐19, and the second round in July–August 2021, following the peak of the wave caused by the delta variant of SARS‐CoV‐2. We used cross‐sectional probability‐to‐size (PPS)‐based multistage cluster sampling to estimate the seroprevalence in the general population of Nepal at the national and provincial levels. We tested for anti‐SARS‐CoV‐2 total antibody using the WANTAI SARS‐CoV‐2 Ab ELISA kit. In Round 1, the overall national seroprevalence was 14.4%, with provincial estimates ranging from 5.3% in Sudurpaschim to 27.3% in Madhesh Province. In Round 2, the estimated national seroprevalence was 70.7%, with the highest in the Madhesh Province (84.8%) and the lowest in the Gandaki Province (62.9%). Seroprevalence was comparable between males and females (Round 1, 15.8% vs. 12.2% and Round 2, 72.3% vs. 68.7%). The seroprevalence in the ecozones—Terai, hills, and mountains—was 76.3%, 65.3%, and 60.5% in Round 2 and 17.7%, 11.7%, and 4.6% in Round 1, respectively. In Nepal, COVID‐19 vaccination was introduced in January 2021. At the peak of the first generalized wave of COVID‐19, most of the population of Nepal remained unexposed to SARS‐CoV‐2. Towards the end of the second generalized wave in April 2021, two thirds of the population was exposed.

## 1 INTRODUCTION

Coronavirus disease (COVID‐19) has become a significant global public health challenge since the first cases were identified in Wuhan, China, in 2019. In January 2020, the World Health Organization (WHO) recognized the severe acute respiratory syndrome coronavirus 2 (SARS‐CoV‐2) outbreak as an international public health emergency, and soon after, in March 2020, the WHO declared a pandemic. In Nepal, the first case of COVID‐19 was detected in January 2020; the patient was a Nepalese student who had recently returned from studying at the Wuhan University of Technology.^1^, ^2^ Since then, Nepal has experienced several generalized waves of COVID‐19, with the first occurring between August and November 2020. A more significant second wave, predominantly attributable to the delta variant, occurred between March and June 2021, concurrently with a similar wave in India.^3^ As of May 23, 2022, the total number of confirmed COVID‐19 cases in Nepal, which had a population of 30 million, was 979,076, with 11,952 deaths.^4^ Laboratory‐confirmed case detection in Nepal has been limited and has only been available in the case of symptomatic individuals and a fraction of their traced contacts. The actual number of cases is presumed to be larger, as the disease was most likely undetected in most asymptomatic cases.

The presence of anti‐SARS‐CoV‐2 antibodies in blood samples can serve as an indicator of previous exposure to the virus, and seroprevalence studies can, therefore, provide insight into the incidence and spread of COVID‐19. Accordingly, the WHO developed the Unity Studies initiative, creating a set of standardized protocols for seroepidemiological research to support the implementation of regional and national seroprevalence studies worldwide.^5^ The WHO recently published a meta‐analysis of early results obtained by the Unity Studies and those of other seroprevalence studies to assess pooled regional and global seroprevalence estimates. The estimated global SARS‐CoV‐2 seroprevalence by April 2021 was 26.1% (95% confidence interval [CI]: 24.6% to 27.6%), with an estimated median seroprevalence‐to‐case ratio of 61.9:1 in lower–middle‐income countries (LMICs). In Southeast Asia, the pooled estimated seroprevalence was 37.5% in the first quarter of 2021 [37.5–37.5], which increased from 29.7% [22.4–37.0] at the end of 2020.^6^ Many countries, including Nepal, began vaccination campaigns in 2021.^7^

To date, few seroprevalence studies have been performed in Nepal, and previous studies were focused on the Katmandu district, the nation's capital and most populated region. A December 2020 study of 800 healthcare workers (HCWs) from 20 hospitals in Katmandu found an adjusted seropositivity rate of 38.17% (95% CI: 29.26–47.82).^3^ A similar study conducted between December 2020 and January 2021 included 185 HCWs from the Manmohan Memorial Medical College and Teaching Hospital and found a seroprevalence of 22%.^1^ To further assess the incidence and spread of COVID‐19 in Nepal, this study aimed to estimate seroprevalence rates and to assess risk factors for infection in the general population of Nepal at the national and provincial levels, in both urban and rural areas, as well as to assess changes in COVID‐19 infection rates over time in the population, by conducting two rounds of sampling at two different timepoints in 2020 and 2021.

## 2 METHODS

### 2.1 Study design and setting

Nepal is a landlocked country surrounded by India and China with three topographical divisions: the northern mountain region, the middle hill region, and the lowland Terai region in southern Nepal.^8^ This division is based on the altitude from sea level. A federated system governs Nepal with several administrative tiers: one federal level, seven provincial level, and 753 local level governments. Additionally, there are 77 districts with their organizational structures distributed among the provinces.^9^ Each local level is divided into wards; a ward is the smallest local government unit and was considered the primary sampling unit in this probability‐to‐size (PPS)‐based multistage cluster sampling study. We aligned the protocol for this study with that proposed by the WHO Unity Studies initiative.^10^

### 2.2 Sampling methods

To calculate the sample, we used the formula for cluster sampling surveys.^11^ The first round of sampling was designed to estimate the SARS‐CoV‐2 seroprevalence at the province level. The expected seroprevalence was approximately 1%, based on surveillance data and the meager impact that COVID‐19 had at the time of planning the first sampling round. The margin of error was set to 1.5% with an alpha error of 5%, and the design effect was assumed to be 2, with a non‐response rate of 10%. The resulting calculated sample size for each province was 372 individuals. Considering the difficult terrain and possible infrastructure issues that could complicate the storage, transport, and processing of collected blood samples, the per‐province sample size was increased to 450 individuals. Thirty municipal ward clusters were randomly selected from each province, amounting to 210 clusters chosen from the seven provinces. Clusters encompassing more than 250 households were first partitioned such that each partition contained fewer than 250 households; subsequently, one partition was selected randomly for sampling from that cluster. Fifteen households were randomly selected from each designated cluster.

Nepal has a difficult terrain due to an uneven distribution of healthcare services, including laboratory facilities and the implementation of the disease surveillance system. Routine surveillance data on COVID‐19 revealed differences in case reporting based on geography. Nonetheless, public health experts understood that these differences were merely due to the uneven distribution of services and believed that seroprevalence was more or less equal around the country. For the second round of sampling, each province was divided into three sampling strata. Stratum 1 included the district with the provincial capital. Strata 2 and 3 were defined based on COVID‐19‐confirmed caseload data by combining the constituent districts according to their proportionate contribution to the total reported caseload in the province. Stratum 2 (high caseload) included districts with higher disease prevalence rates (>10%), and Stratum 3 (low caseload) had districts with lower prevalence rates. In the case of Bagmati Province, an additional stratum was added to include the Kathmandu, Lalitpur, and Bhaktapur districts (formally known as the Kathmandu Valley), amounting to 22 strata. Separate sample sizes were calculated for each provincial stratum based on the projected seroprevalence rates using the results from the first round of sampling. The projected seroprevalence rates for all the provinces were above 50% as of February 1, 2021. Therefore, a seroprevalence rate of 50% was used to derive the maximum sample size, with a precision of 40% for the seroprevalence point estimate. Design effect two was used to account for the chosen sampling technique. The total calculated sample size was 13,710 participants from 1370 municipal ward clusters, with 10 households from each cluster, taking into account a 10% non‐response rate. The ratio between the urban and rural clusters within the sampling frame was also maintained in the sample size calculation. The Central Bureau of Statistics of Nepal reviewed and agreed with the sample size.

### 2.3 Participant recruitment

In each designated household, one individual was randomly selected from the eligible household members using a modified Kish Grid^12^ to avoid selection bias. Eligible participants for both surveys included household members aged 6 months or older who had been living in Nepal for at least 4 weeks before the blood sampling date. Household members who were unwilling to provide informed consent and those with contraindications to venipuncture were excluded from the study.

### 2.4 Survey data and blood sample collection

To collect survey and sample data, field teams of five members each were formed, comprising a coordinator for overall supervision and coordination, an enumerator for survey data collection, a phlebotomist for blood sample collection, a female community health volunteer, and a ward official or representative to guide the survey team through the selected cluster. We implemented several monitoring mechanisms to ensure the overall quality of data collection, including rechecking the geo‐coordinates of selected households and verifying the tracking process of the transfer of each sample from the collection site to the National Public Health Laboratory (NPHL). Throughout the study, monitoring teams that included high‐level officials from the Ministry of Health and Population, local leaders, and WHO representatives performed a detailed supervision of the fieldwork.

Trained enumerators adhered to Infection Prevention and Control (IPC) measures during the data collection. All participants provided informed consent. For minor participants, the caregiver/legal guardian provided consent. Following informed consent, data were collected using a structured questionnaire implemented with the help of computer‐aided personal interviewing (CAPI) software. The main areas covered by the questionnaire included demographic details, COVID‐19 status, clinical features, comorbidities, COVID‐19 vaccination status, and international travel history. A trained phlebotomist collected the blood samples. All samples were immediately transferred to the NPHL using a reverse cold chain system.

### 2.5 Serological testing

Serum samples were tested for the presence of SARS‐CoV‐2‐specific total antibodies (immunoglobulin (Ig)M and IgA) in a Biosafety Level 2 laboratory at the NPHL using an enzyme‐linked immunoassay (ELISA) kit (WANTAI SARS‐CoV‐2 Ab ELISA kit, Wantai Diagnostics) according to the manufacturer's protocol. This assay is used in all countries participating in the WHO Unity Studies.^5^ NPHL, Nepal tested a National Institute for Biological Standards and Control (NIBSC) anti‐SARS‐CoV‐2 sera panel obtained through the WHO was used as control and was tested along with the samples.^13^

### 2.6 Data analysis

Survey design weights were calculated using selection probabilities from the multiple sampling stages. Given the low non‐response rate, we did not adjust weights for non‐response. Survey weights were post‐stratified using province population values to estimate the population totals. We calculated the national‐level seroprevalence of SARS‐CoV‐2 antibodies. Similarly, seroprevalence was determined in different strata (i.e., provincial, sex, age groups, and ecozones). We used the chi‐square test to determine statistically significant differences in the reported seroprevalence. We used odds ratios to identify probable risk factors among the study participants. All important crude odds ratios were subjected to logistic regression analysis accounting for the survey weights. We considered differences statistically significant at a *p* value of 0.05. Statistical analyses were performed using the base version of R (version 4.0.2, which included survey 4.1‐1, epiR 1.0‐15, and epitools 0.5‐10.1). A confidence level of 95% was used for the interval estimates.

## 3 RESULTS

### 3.1 Demographic data

**FIGURE 1:**
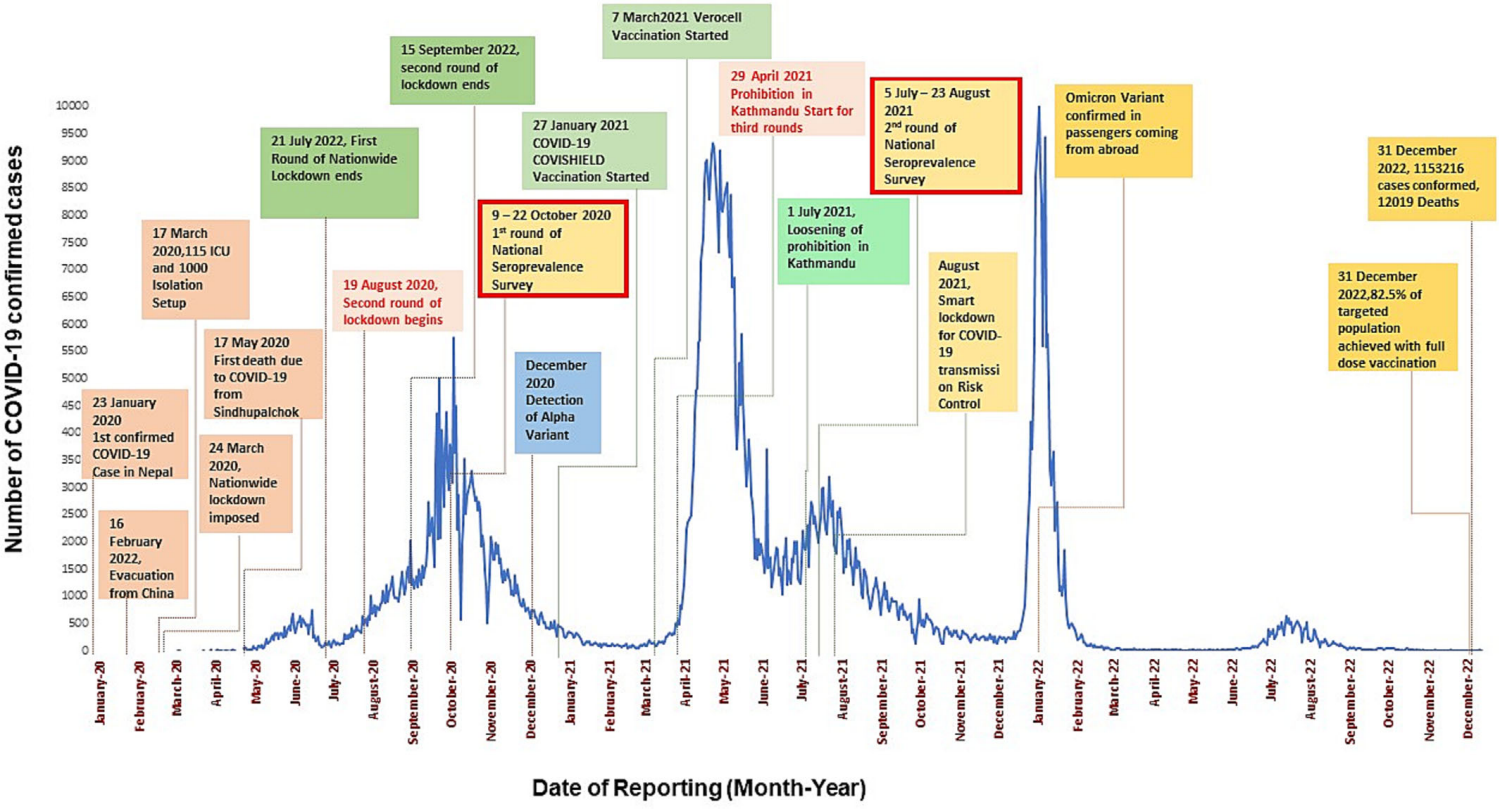
Epidemic curve for COVID‐19, Nepal, 2020–2022.

**FIGURE 2:**
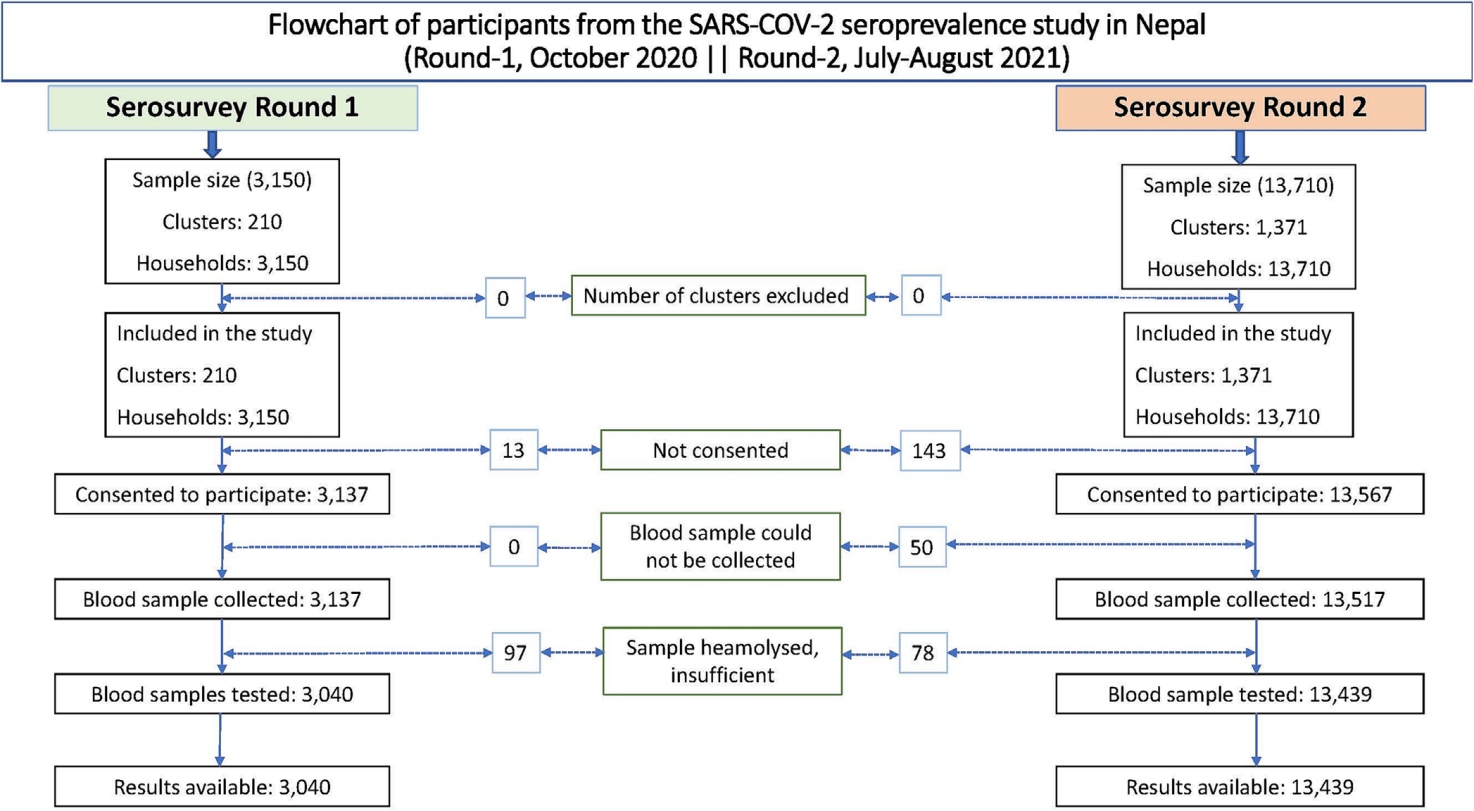
Flowchart of participants from the SARS‐CoV‐2 seroprevalence study in Nepal (Survey 1, October 2020; Survey 2, July–August 2021).

The first round of sampling was performed between October 9 and October 22, 2020. The intended number of participants for data and blood sample collection was 3150 from 3150 households distributed among 210 clusters across Nepal; however, the analysis included only 3040 complete records (Figures 1 and 2). The second round of sampling was performed between July 5 and August 14, 2021. For the second round, the intended number of participants for data and blood sample collection was 13,710 from 1371 geographic clusters; however, the analysis included only 13,439 complete records (Figures 1 and 2). Men outnumbered women in participation in both rounds of serosurveys (57% in Round 1 and 53% in Round 2). Most of the participants (72% in Round 1 and 74% in Round 2) were between the ages of 5 and 65 years. In both rounds of sampling, the ≥85‐year‐old age group was the smallest (0.8% and 0.5%). The vaccination campaign had not yet begun during the first round of sampling. At the time of sample collection during Round 2, 7.3% of participants were fully vaccinated, 15% had received one dose, and 77.8% had not been vaccinated (Table 1).

**TABLE 1:** Distribution of SARS‐CoV‐2 seroprevalence with COVID‐19 vaccination status by serosurvey, Nepal, 2020–2021.

	First survey *n*1 = 3040			Second survey *n*2 = 13,439		
	Number tested	Number positive (%)	Adjusted prevalence (95% CI)	Number tested	Number positive (%)	Adjusted prevalence (95% CI)
Fully vaccinated	NA	NA	NA	978	877 (89.7)	89.1 (86.0–91.6)
Partially vaccinated	NA	NA	NA	2021	1621 (80.2)	82.0 (79.8–84.0)
Not vaccinated	NA	NA	NA	10,440	6655 (63.7)	66.5 (65.1–67.8)

### 3.2 Overall seroprevalence

In Round 1, blood samples were collected from 3137 participants (13 responded to the survey but did not consent to blood sampling). Of these, 97 samples were unavailable for laboratory testing because they were hemolyzed or the quantity was insufficient for ELISA testing. The remaining 3040 samples were analyzed (Figure 2). The 362 (11.9%) of the 3040 samples collected during Round 1 tested positive for SARS‐CoV‐2 antibodies. The overall weighted seroprevalence in Round 1 was 14.4% (CI: 11.8, 17.0). Of the 97 participants who underwent polymerase chain reaction (PCR) or rapid diagnostic testing, 4.1% tested positive for SARS‐CoV‐2 (Table 2).

**TABLE 2:** National and provincial level SARS‐CoV‐2 seroprevalence by serosurvey, Nepal, 2020–2021.

	First survey *n*1 = 3040			Second survey *n*2 = 13,439			*p* value
	Number tested	Number positive (%)	Adjusted prevalence (95% CI)	Number tested	Number positive (%)	Adjusted prevalence (95% CI)	
Province							
Koshi	442	44 (10.0)	7.8 (4.5–13.1)	1474	931 (63.2)	63.1 (60.0–66.1)	<0.000
Madhesh	438	114 (26.0)	27.3 (21.0–34.8)	1403	1200 (85.5)	84.8 (82.1–87.1)	<0.000
Bagmati	431	79 (18.3)	20.7 (12.1–33.0)	1917	1318 (68.8)	70.0 (66.6–73.2)	<0.000
Gandaki	438	36 (8.2)	8.8 (4.7–15.8)	1872	1183 (63.2)	63.9 (61.1–66.7)	<0.000
Lumbini	419	32 (7.6)	7.4 (5.1–10.7)	2003	1463 (73.0)	72.2 (69.7–74.5)	<0.000
Karnali	429	31 (7.2)	7.6 (4.8–11.9)	2091	1325 (63.4)	62.9 (59.9–65.8)	<0.000
Sudurpaschim	443	26 (5.9)	5.3 (3.0–8.9)	2679	1733 (64.7)	64.6 (62.1–67.0)	<0.000
National							
Nepal	3040	362 (11.9)	14.4 (11.8–17.0)	13,439	9153 (68.1)	70.7 (69.6–72.0)	<0.000

In Round 2, blood samples were collected from 13,517 participants (50 participants responded to the survey but did not consent to blood sampling, while 143 did not consent to participation in the survey). Of these samples, 78 samples were not available for laboratory testing because they were hemolyzed or the quantity was insufficient for ELISA testing. The remaining 13,439 samples were analyzed (Figure 2). A total of 9153 (68.1%) of the 13,439 samples collected in Round 2 tested positive for SARS‐CoV‐2 antibodies. The overall weighted seroprevalence in Round 2 was 70.7% (CI: 69.6, 72.0). Of the 771 participants who underwent PCR or rapid diagnostic testing, 36.2% tested positive for SARS‐CoV‐2 in routine diagnostic tests conducted independently of this study (Table 2).

### 3.3 Seroprevalence by age and sex

In Round 1, male participants had a weighted seroprevalence rate of 15.8% (CI: 13.0–19.1), which was higher than that of female participants (12.2%; CI: 9.0–16.4). The 35–44‐year‐old age group had the highest positivity rate (19.5%; CI: 14.4–25.9), while the >75‐ and <5‐year‐old age groups had the lowest rates (Table 3).

**TABLE 3:** Distribution of SARS‐CoV‐2 seroprevalence among sex by serosurvey, Nepal, 2020–2021.

	First survey *n*1 = 3040			Second survey *n*2 = 13,439			*p* value
	Number tested	Number positive (%)	Adjusted prevalence (95% CI)	Number tested	Number positive (%)	Adjusted prevalence (95% CI)	
Male	1744	232 (13.3)	15.8 (13–19.1)	7187	5008 (69.7)	72.3 (70.8–73.7)	<0.000
Female	1296	130 (10.0)	12.2 (9.0–16.4)	6252	4145 (66.3)	68.7 (67.0–70.4)	<0.000

Like in Round 1, male participants had a higher weighted seroprevalence than female participants in Round 2 (72.3%; CI: 70.8–73.7 vs. 68.7%; CI: 67.0–70.4). In Round 2, the 65–74‐year‐old age group had the highest positivity rate (79.4%; CI: 75.8–82.6). Unlike in Round 1, the ≥85‐year‐old age group also had high seroprevalence (73.7%; CI: 60.3–83.7), while the rates in children under 5 years (56.2%; CI: 48.0–64.0) and in those aged 5–14 years (58.8%; CI: 55.0–62.4) were lower (Table 4).

**TABLE 4:** Distribution of SARS‐CoV‐2 seroprevalence among age groups by serosurvey, Nepal, 2020–2021.

Age group (years)	First survey *n*1 = 3040			Second survey *n*2 = 13,439			*p* value
	Number tested	Number positive (%)	Adjusted prevalence (95% CI)	Number tested	Number positive (%)	Adjusted prevalence (95% CI)	
0–4	110	9 (8.2)	9.5 (4.6–18.6)	221	124 (56.1)	56.2 (48.0–64.0)	<0.000
5–14	345	26 (7.5)	8.5 (5.4–13.3)	1242	675 (54.3)	58.8 (55.0–62.4)	<0.000
15–24	547	61 (11.2)	14.3 (10.1–19.8)	1936	1302 (67.3)	69.6 (66.4–72.6)	<0.000
25–34	463	61 (13.2)	13.9 (10.5–18.1)	2216	1507 (68.0)	70.7 (67.9–73.4)	<0.000
35–44	487	79 (16.2)	19.5 (14.4–25.9)	2342	1644 (70.2)	73.3 (70.9–75.6)	<0.000
45–54	428	53 (12.4)	16.0 (10.9–22.9)	2216	1499 (67.6)	71.1 (68.4–73.6)	<0.000
55–64	350	45 (12.9)	17.0 (12.0–23.6)	1686	1181 (70.0)	73.9 (71.0–76.6)	<0.000
65–74	208	24 (11.5)	16.2 (10.3–24.7)	1087	850 (78.2)	79.4 (75.8–82.6)	<0.000
75–84	77	3 (3.9)	6.0 (1.6–20.5)	418	318 (76.1)	78.6 (73.5–83.0)	<0.000
≥85	25	1 (4)	2.1 (0.2–15.3)	75	53 (70.7)	73.7 (60.3–83.7)	<0.000

### 3.4 Seroprevalence results by geographical region

In Round 1, seroprevalence rates were the highest in the Terai ecological zone (17.7%; CI: 14.7–21.2) compared with the hill (11.7%; CI: 7.5–17.9) and mountain zones (4.6%; CI: 2.2–9.5). Madhesh had the highest weighted seroprevalence (27.3%; CI: 21.0–34.8), and Sudurpaschim had the lowest (5.3%; CI: 3.0–8.9) (Table 2). The Terai region had the highest seroprevalence rate in Round 2 as well (76.3%; CI: 74.8–77.7) (Table 5). Urban areas had a slightly higher prevalence rate than rural areas (71.8%; CI: 70.2–73.3 vs. 68.6%; CI: 66.5–70.6) (Table 6). Madhesh still had the highest prevalence rate among the seven provinces (84.8%; CI: 82.1–87.1), and Karnali had the lowest (62.9%; CI: 59.9–65.8) (Table 2).

**TABLE 5:** Distribution of SARS‐CoV‐2 seroprevalence among ecological zones by serosurvey, Nepal, 2020–2021.

Ecological zone	First survey *n*1 = 3040			Second survey *n*2 = 13,439			*p* value
	Number tested	Number positive (%)	Adjusted prevalence (95% CI)	Number tested	Number positive (%)	Adjusted prevalence (95% CI)	
Terai	1312	222 (16.9)	17.7 (14.7–21.2)	6167	4559 (73.9)	76.3 (74.8–77.7)	<0.000
Hill	1477	128 (8.7)	11.7 (7.5–17.9)	6270	3984 (63.5)	65.3 (63.4–67.3)	<0.000
Mountain	251	12 (4.8)	4.6 (2.2–9.5)	1002	610 (60.9)	60.5 (55.2–65.6)	<0.000

**TABLE 6:** Distribution of SARS‐CoV‐2 Seroprevalence by urban–rural areas, Nepal, 2020–2021.

Residence	First survey *n*1 = 3040			Second survey *n*2 = 13,439		
	Number tested	Number positive (%)	Adjusted prevalence (95% CI)	Number tested	Number positive (%)	Adjusted prevalence (95% CI)
Urban		‐	‐	8539	5879 (68.8)	71.8 (70.2–73.3)
Rural		‐	‐	4900	3274 (66.8)	68.6 (66.5–70.6)

### 3.5 Seroprevalence and vaccination

In Round 1, vaccination was yet to be available in Nepal. In Round 2, 22% of participants were fully or partially vaccinated. The weighted seroprevalence rate was 89.1% (CI: 86.0–91.6) in the fully vaccinated group and 82% (CI: 79.8–84.0) in the partially vaccinated group. The seroprevalence rate in the unvaccinated group was 66.5% (CI: 65.1–67.8) (Table 1).

### 3.6 Estimated infection‐to‐case ratio

During Round 1, the total number of confirmed cases in Nepal was 172,800 (October 15, 2020). Based on the estimated national seroprevalence determined in Round 1 (14.4%), the estimated true number of SARS‐CoV‐2 infections at the time of Round 1 was 4,320,000, with an infection‐to‐case detection ratio of 25:1. The total number of confirmed cases in Nepal had increased to 742,817 by the time of Round 2. Based on the estimated national seroprevalence rate of 70.7% determined during Round 2, the estimated actual number of infections was 21,210,000 at that time, with an infection‐to‐case detection ratio of 29:1. (Table 7).

**TABLE 7:** Infection‐to‐case ratio in two rounds of SARS‐CoV‐2 seroprevalence surveys, Nepal, 2020–2021.

	First serosurvey		Second serosurvey	
	Number (October 15, 2020)	Percent^a^	Number (July 27, 2021)	Percent^a^
Reported cases (RT‐PCR)	172,800	0.6	685,673	2.3
Reported cases (RT‐PCR and Ag RDTs)			742,817	2.5
Estimated infections^b^	4,320,000	14.4 (95% CI: 11.8–17.0)	21,210,000	70.7 (95% CI: 69.6–72.0)
Infection‐to‐case ratio	25:1		29:1	

^a^ Percent of total population of 30 million.

^b^ Estimated infection was derived by multiplying point estimate of seroprevalence by total population.

### 3.7 Risk factors for infection

In Round 1, the following factors were significantly associated with seroprevalence: age (children vs. working age/older adults) and living in the Terai zone (Terai zone vs. mountain zone). In Round 2 as well, age (children) was significantly associated with seroprevalence, as were the male sex and living in the Terai zone (Table 8). We also estimated the SARS‐CoV‐2 infection risk based on sex, urban–rural setting, income, and ethnicity (Table 8).

**TABLE 8:** Risk factor assessment for SARS‐CoV‐2 infection by serosurvey, Nepal, 2020–2021.

First survey			Second survey	
Risk factor	OR (95% CI)	*p* value	OR (95% CI)	*p* value
Age				
Children	0.51 (0.34–0.80)	0.002	0.55 (0.47–0.63)	<0.001
Elderly	0.76 (0.47–1.23)	0.27	1.43 (1.20–1.70)	<0.001
Working	Reference		Reference	
Sex				
Male	1.22 (0.89–1.67)	0.23	1.17 (1.06–1.30)	0.002
Female	Reference		Reference	
Ecozones				
Terai	4.31 (1.97–9.42)	<0.001	2.05 (1.60–2.63)	<0.001
Hills	2.91 (1.22–6.91)	0.02	1.19 (0.93–1.52)	0.163
Mountains	Reference		Reference	
Urban–rural				
Urban	‐	‐	1.17 (0.94–1.22)	0.282
Rural	‐	‐	Reference	
Income (NPR/month)				
>10,000	1.89 (1.31–2.72)	<0.001	‐	‐
0–10,000	Reference		‐	‐
Ethnic‐caste groups				
Dalit	1.47 (0.96–2.23)	0.08	‐	‐
Disadvantaged Janajati	1.22 (0.78–1.91)	0.38	‐	‐
Disadvantaged non‐Dalit Terai caste groups	2.74 (1.75–4.29)	<0.001	‐	‐
Relatively advantaged Janajatis	2.63 (1.48–4.68)	0.001	‐	‐
Others, foreigners, and data missing	1.10 (0.63–1.90)	0.74	‐	‐
Upper caste groups	Reference		‐	‐

## 4 DISCUSSION

Based on the data collected during the two rounds of sampling, COVID‐19 spread rapidly throughout Nepal between October 2020 and August 2021, such that an estimated 70% of Nepal's population was exposed to SARS‐CoV‐2 by the end of the study period. These results, which were based on samples collected from all the provinces in Nepal located across both urban and rural areas, indicate that the true number of COVID‐19 cases was much larger than the number of reported confirmed cases during this period, suggesting that surveillance measures failed to capture the extent and spread of the pandemic, as has been reported previously.^14^ Seroprevalence rates were the highest in older adults during the second round of sampling, which may result from vaccination efforts targeted at older adults in early 2021.

Surveillance data obtained during both sampling periods (October 2020 and August 2021) indicated that a more significant proportion of males (70% of the total number of infected individuals in October 2020 and 60% in August 2021) than females were affected.^15^, ^16^ However, in both rounds of sampling, we found similar seropositivity rates between men and women. In Round 1, the seroprevalence in male and female participants was 15.8% and 12.2%, respectively, and the difference was not significant (*p* = 0.07). In Round 2, the male sex was associated with a slightly higher, considerable risk of infection. The difference between our findings and those based on national surveillance data may indicate a gap in testing or equity challenges in accessing COVID‐19 testing in the case of women; this highlights the need for serious attention from federal and provincial health authorities, as well as policymakers.

Regarding age, our results align with findings based on contemporary surveillance data, suggesting that the most affected age groups were the most economically productive. In October 2020, 85.6% of confirmed cases among men involved those of working age (15–54 years).^16^ In our first round of sampling, 70.1% of seropositive cases (men and women) involved individuals of this age group. Surveillance data from August 2021 indicate that men between the ages of 15–54 accounted for 78% of cases,^15^ and we found a similar rate (72%) in participants in this age range (men and women) during the second round of sampling. Younger adults and workers tend to be more mobile and less adherent to public health and social measures. They may have a more significant impact on disease transmission in the community than the elderly population, which has been the focus of public health measures. We found a substantial increase in seroprevalence among elderly participants (≥75 years) between the first and second rounds of sampling.

The school‐age population or children below 15 exhibited comparatively lower seroprevalence rates during the first and second rounds (7.7% and 54.6%, respectively; *p* < 0.0001); these rates were higher than those observed based on the surveillance data.^15^, ^16^ The lower detection rate among this population may be due to a more significant number of cases of asymptomatic or mild disease in this group. A recent WHO report indicates the unrecognized impact of the pandemic in terms of excess deaths. In Nepal, there were an estimated 83 excess deaths per 100,000 people in 2021, which was lower than that in many other countries, such as India (171/100,000) and the United States (140/100,000).^17^ The relatively young population in Nepal has been proposed as a factor that contributed to the comparatively lower death rates in the country, as older age is associated with more severe disease and higher mortality.^3^

The seroprevalence rates varied widely between provinces in the first round of sampling, ranging from 5.3% in Sudurpaschim to 27.3% in Madhesh Province. This distribution was somewhat unexpected, as the highest seroprevalence was expected in the Bagmati Province. Approximately 35% of confirmed cases in Nepal were identified in the Kathmandu Valley area in Bagmati Province (October 2020).^16^ However, the highest seroprevalence (27.3%) was found in Madhesh Province in our study, which may indicate a surveillance and/or testing gap in this region that should be investigated and addressed by the provincial and federal governments. In the second round, the highest seroprevalence (84.8%) was again found in Madhesh Province, whereas the lowest was seen in the Karnali Province (62.9%).

Survey data collected during both rounds revealed lower seroprevalence rates in the easternmost and westernmost provinces of the country, including Koshi Province in the east and Sudurpaschim and Karnali in the west. These areas are known for their relatively difficult geographical accessibility. However, the epidemiologic curves for Sudurpaschim and Karnali Provinces show multiple smaller peaks throughout 2020, suggesting repeated point source exposures and reintroduction of the disease.^18^ This is likely due to travelers from outside the country entering Nepal through border crossings. This factor should be considered by provincial health authorities when establishing public health measures in these regions.

Nepal's terrain can be grouped into three ecological zones that correlate with the elevation from sea level: Terai plains, hill regions, and mountain regions. In the first round of sampling, the seroprevalence rates in the Terai and hill regions were significantly higher than those in the mountain regions. In this round, the risk of infection in the Terai and hill regions was approximately four and three times higher than that in the mountain regions. These differences were somewhat tempered in the second round of sampling, in which the difference remained significant only in the case of the Terai zone, where residents had double the risk of infection than that in the mountain regions. However, in both rounds of sampling, seroprevalence rates consistently decreased as the elevation from sea level increased. This observation may primarily be due to population density, geographic accessibility constraints, and the closure of major tourist locations. Still, it may also indicate the presence of regional variations in factors related to COVID‐19 prevention and control.

Higher income was also associated with infection, with those earning >10,000 Nepalese rupees per month having twice the risk of earning less. This observation was unexpected, as socioeconomically disadvantaged individuals may have less ability to isolate and access vaccination and other preventive measures.^19^, ^20^ This finding highlights the need for further research to understand the regional role of Nepal within Southeast Asia, where income may be closely linked to mobility and migration within Nepal and abroad.^14^, ^21^ We found that marginalized or socioeconomically disadvantaged groups, particularly the indigenous Terai caste and Janajati groups,^22^ have approximately twice the risk of contracting SARS‐CoV‐2 infection than the Dalit group does. This finding indicates that COVID‐19 risk is associated with socioeconomic status, which has been observed in other countries as well.^23^, ^24^, ^25^

Frontline workers (HCWs and security personnel) exhibited lower seroprevalence rates than most other occupation groups surveyed in our study (all except agriculture) in the first round of sampling before vaccination was available. This may reflect the effective use of public health and social measures by this group. Two other studies conducted several months later, in December 2020 and January 2021, found higher positivity rates in HCWs^1^, ^3^; however, these studies were conducted in Kathmandu only. Paudel et al. found that 34% of HCWs reported asymptomatic infections.^3^ By the second round of sampling in our study, conducted after vaccination became available, seroprevalence rates had greatly increased in the frontline worker group (89%). Nepal's vaccination campaign began in early 2021 in individuals over the age of ≥18 years, and during the initial stage of the campaign, health and frontline workers and individuals older than 65 years were prioritized.^3^, ^7^ By the time the second round of sampling was conducted in this study, an estimated 16% of the population had received at least one vaccination dose and 13% were fully vaccinated.^26^ Around 22% of the participants in our study reported being partially or fully vaccinated. The fully vaccinated group had the highest seroprevalence rate (89%), followed by the partially vaccinated (82%) and unvaccinated (67%) groups. Possible reasons why the fully vaccinated group did not acquire 100% immunity, may be multifactorial, and may include primary and secondary vaccine failure.^27^

We compared our results with other published SARS‐CoV‐2 serosurveys conducted using the UNITY protocol. One study conducted in October–November 2020 in Pakistan also found a low prevalence (7.1%). The prevalence distribution over age and sex groups was also comparable^28^ with our findings. Our results show some similarities and differences with the SARS‐CoV‐2 systematic review and meta‐analysis conducted by Bergeri et al. Our first serosurvey suggested a 25:1 infection detection ratio in the last quarter of 2020, while meta‐analyzed data suggested a 40:1 ratio in the Southeast Asia Region (SEAR). Similarly, seroprevalence for SARS‐CoV‐2 at the national level was less than the overall reported for the SEAR (14% vs. 25%).^29^ However, the situation was more comparable by the third quarter of 2021 (71% vs. 75%), indicating the high transmission during the delta wave while the vaccination campaign was also rolling out. This may indicate the lapses in adherence to public health and social measures that were in place to curtail viral transmission and relatively high vaccination coverage. A SARS‐CoV‐2 serosurvey conducted in Togo reported high seroprevalence among urban dwellers, young adults (30–49 years), and vaccinated individuals, closely matching our findings.^30^ It is important to note that our study used the WANTAI SARS‐CoV‐2 Ab ELISA for total antibodies to SARS‐CoV‐2 as recommended by Unity Studies protocol^5^ to ensure comparability of results from different settings.

Our study has several limitations. There were differences between the proportions of individuals based on age and sex between the sampled population and the actual population, which may have resulted from selection bias caused by deviation from the study protocol. Specifically, our sample included a more significant proportion of male participants than female participants and a larger proportion of older people than that based on the national distribution. Additionally, we relied on self‐reported demographic and vaccination status‐related data. The Wantai SARS‐CoV‐2 ELISA kit only measures total antibodies. It does not provide information regarding the relative levels of IgM and IgG, and we did not account for waning antibody levels from previous infections. This study also has many strengths, particularly the probability sampling method and inclusion of participants from all seven provinces. We used a standardized protocol developed by the WHO and validated assay developed for the Unity Studies.

## 5 CONCLUSION

The results obtained by this study, which is the first national seroprevalence study in Nepal, suggest that a large proportion of the population of Nepal was exposed to SARS‐CoV‐2 by August 2021 and that widespread transmission occurred throughout all seven provinces between October 2020 and August 2021. However, these results demonstrate that the rate of COVID‐19 detection during this period was low and did not reflect the actual rate of infection. Additionally, the heterogeneous transmission patterns across different populations and geographic zones suggest variable disease transmission dynamics. A substantial proportion of the population of Nepal is still susceptible to COVID‐19, particularly in the hill and mountain regions. Surveillance measures, including rapid laboratory turnaround, should target areas where higher transmission may occur, particularly in the Terai zone, and further investigation into the patterns of COVID‐19 transmission by region should be performed.

## AUTHOR CONTRIBUTIONS

**Krishna Prasad Paudel:** Conceptualization and implementation—lead editing—equal contribution. **Reuben Samuel:** Conceptualization and implementation—lead editing—equal contribution. **Runa Jha:** Laboratory data collection—lead. **Basu Dev Pandey:** Conceptualization; study design. **Chathura Edirisuriya:** Epidemiological study design and data analysis and writing the first draft of the manuscript. **Nebin Lal Shrestha:** Study design. **Pradip Gyawali:** Study design. **Amrit Pokhrel:** Epidemiological data collection; analysis. **Lilee Shrestha:** Laboratory testing and data collection. **Ram Kumar Mahato:** Epidemiology data collection; monitoring. **Shaikh Shah Hossain:** Epidemiology study design; monitoring; analysis; writing. **Govindakarnavar Arunkumar:** Laboratory methods; data collection; analysis; writing; review. **Anindya Sekhar Bose:** Epidemiological study design. **Meghnath Dhimal:** Epidemiological study design. **Dipendra Gautam:** Epidemiological data collection; monitoring; review. **Subash Neupane:** Coordination of field data collection; monitoring; review. **Nishant Thakur:** Epidemiological data collection; monitoring. **Saugat Shrestha:** Coordination of sample collection and laboratory data collection. **Nirajan Bhusal:** Laboratory testing and data collection; analysis. **Priya Jha:** Laboratory testing and data collection; analysis. **Binod Prasad Gupta:** Field coordination and monitoring of implementation. **Rajan Bikram Rayamajhi:** Field coordination and monitoring of implementation. **Koshal Chandra Subedi:** Epidemiological data collection and monitoring. **Shashi Kandel:** Field monitoring.^1^ Mukesh Poudel: Field monitoring.^1^ **Lila Bikram Thapa:** Field monitoring.^1^ **Guna Nidhi Sharma:** Field monitoring. **Allison Eugenio Gocotano:** Coordination of implementation; review. **Avinash K. Sunny:** Field data collection tool; data collection; analysis. **Rabin Gautam:** Field data collection tool; data collection; analysis. **Deepak Raj Bhatta:** Field monitoring. **Bal Krishna Awale:** Laboratory tests; data collection analysis. **Bhola Roka:** Field data collection and monitoring.^1^ **Hemant Chandra Ojha:** Field monitoring. **Phanindra Baral:** Filed Monitoring. **Mahendra Dhose Adhikari:** Field monitoring. **Guna Raj Lohani:** Review. **Mahendra Shrestha:** Review.^6^ **Dipendra Raman Singh:** Review. **Laxman Aryal:** Review. **Rajesh Sambhajirao Pandav:** Review. **Roshan Pokhrel:** Review.

## CONFLICT OF INTEREST STATEMENT

The authors have no relevant conflicts of interest to report.

### PEER REVIEW

The peer review history for this article is available at https://www.webofscience.com/api/gateway/wos/peer-review/10.1111/irv.13234.

## ETHICS STATEMENT

The National Health Research Council (NHRC) provided the ethical approval (NHRC Ref No. 934, dated October 6, 2020, and NHRC Ref No. 3546, dated June 4, 2021). All participants provided informed consent.

## DISCLAIMER

This work represents the personal opinion of the authors and not that of the World Health Organization, Ministry of Health and Population, Nepal and the Institutions they belong to.

## Data Availability

The data that support the findings of this study are available in the manuscript, and additional data are available from the corresponding author upon reasonable request.
